# A short term comparative evaluation of the efficacy of diode laser with desensitizing toothpastes and mouthwashes in the treatment of dentinal hypersensitivity

**DOI:** 10.4317/jced.59063

**Published:** 2022-03-01

**Authors:** Anirudh B. Acharya, Apoorva Chandrashekar, Srinath Thakur

**Affiliations:** 1 Department of Preventive and Restorative Dentistry, College of Dental Medicine, University of Sharjah, Sharjah, U.A.E; 2 Department of Periodontics, AJ Shetty Institute of Dental Sciences, Mangaluru-575004, Karnataka, India; 3 Department of Periodontics, AJ Shetty Institute of Dental Sciences, Mangaluru-575004, Karnataka, India

## Abstract

**Background:**

Treatment of dentinal hypersensitivity (DH) has always been challenging with a wide variety of therapeutic options, in-office and home care. The study objective was to compare the clinical efficacy of diode laser [DL] with four commercially available desensitizing agents (two toothpastes and two mouthwashes) in the treatment of DH.

**Material and Methods:**

This study involved 75 participants (25-45 years) who were categorized as Group 1 (n=15) treated with DL, and Groups 2, 3,4 and 5 (n=15 in each) who were prescribed 3% potassium nitrate mouth rinse, a herbal mouth rinse, potassium nitrate tooth paste and a herbal tooth paste, respectively. DH was assessed by air blast stimulation using air blast syringe, and evaluation of DH was done based on the patient’s subjective response using the Visual Analog Scale (VAS) at baseline, 1 week and 1 month.

**Results:**

DL and the desensitizing agents showed significant reduction in DH at 1 month when compared with baseline, except potassium nitrate mouth wash. However, DL showed more percentage reduction in DH when compared with potassium nitrate tooth paste, herbal mouth wash and paste.

**Conclusions:**

The study implies that even though all five groups showed improvement in terms of DH reduction, DL showed the best results among all the groups.

** Key words:**Dentinal Hypersensitivity, Diode Laser, Potassium Nitrate, Herbal, Visual analog scale.

## Introduction

Dentinal hypersensitivity (DH) is a painful response of the tooth to different stimuli such as ‘brushing, acid diets, and thermal changes’ (1). DH is one of the frequently encountered symptoms in the dental office. The management of DH poses a challenge as it is not well understood. DH is defined as, “a short sharp pain arising from exposed dentin in response to stimuli, typically thermal, evaporative, tactile, osmotic, or chemical and which cannot ascribed to any other dental defect or pathology” (2).

The hydrodynamic theory supported by Branstrom’s evidence is an acceptable explanation of the etiologic mechanisms of DH, upon which is based several treatment options (3,4). These include home-use, over the counter desensitizing mouthwashes, dentifrices and in-office procedures and recently, the dental laser has made inroads in the treatment of DH. Before choosing the DH treatment option, the clinician needs to consider an exclusive differential diagnoses (5).

The home use products are simple, logical and routine options for most realistic and mild to moderate DH. Desensitizing dentifrices and mouthwashes are generally acceptable based on their ready availability and ease of use with daily tooth brushing oral hygiene habits (6). The active ingredients of desensitizing toothpastes and mouthrinses diminish dentinal tubule diameter by precipitation of crystals that makes it effective. Of these, potassium nitrate introduced by Hodosh (7) and accepted by the ADA in 1986 is one of the most frequently used desensitizing agents for the treatment of DH. Potassium ions are thought to act by blocking the action potential generated in intradental nerves (8,9). But there has been a question whether its optimal effectiveness is better through a dentifrice or mouthwash (10), and no strong evidence regarding the efficacy of potassium nitrate formulations as noted in a Cochrane database systematic review (11).

Herbal based toothpastes and mouthwashes have been found effective in the control of plaque and gingivitis (11), and prevention of dental caries (12,13). Phytocomplexes from rhubarb stalks [Rhubarb rhaponicum] and spinach leaves [Spinacia oleracia] reduced dentinal permeability by occluding dentinal tubules by forming calcium oxalate crystals (14), and have been used in different formulations for treating DH.

With the introduction of laser technology and its proven utility in dentistry, a supplementary therapeutic for DH is available. Low output power [He‑Ne or diode lasers] and middle output power [Diode, Nd: YAG, Er:YAG, ErCr:YSGG, CO2, Argon, and potassium titanyl phosphate {KTP} lasers] have been employed in DH treatment (15-18). New diode lasers with higher power output and wavelengths were developed to penetrate tissues with minimal damage. Diode lasers are variants of gallium: aluminium: arsenide [Ga: Al: As; near infrared spectrum;780, 830, and 900 nm; power output from 20-100 mW], or indium: gallium: arsenide: phosphorus [In:Ga:As:P; red spectrum of visible light; 600‑680 nm; power output from 1-50 mW], with a report that lasers may now provide dependable treatment of DH (19,20).

Owing to divergent claims about DH therapy, the present study aimed to compare the efficacy of diode laser [940 nm] with commercially available desensitizing toothpastes and mouthwashes-potassium nitrate and herbal, containing Spinacia oleracea, respectively, for a period of one month.

## Material and Methods

This study was conducted in the Department of Periodontics of the concerned institution in accordance with the Declaration of Helsinki and Guidelines for Good Clinical Practice, after obtaining an ethical clearance from the institution’s ethical committee. Subjects were recruited from the outpatient section and informed consent was secured from the prospective participants. The study duration was 1 month, in which sensitivity scores were measured at baseline, at 1 week and at 1 month.

Inclusion criteria were: patients having at least two sensitive permanent tooth surfaces [buccal/facial aspects of incisors, canines, or premolars]; sensitive tooth surfaces had wasting diseases and/or gingival recession and no history of periodontal therapy in the previous year. Exclusion criteria were: currently undergoing desensitizing treatment; medical [including psychiatric and pharmacotherapeutic] history that could compromise the study protocol; pregnant and/or lactating women; any known allergies/history of allergies to dentifrice contents; systemic conditions that are etiologic or predisposing to dentinal hypersensitivity; eating disorders; any dental treatment that might affect the desensitizing agent being used; any other pathology.

Seventy-five systemically healthy volunteers between 25 to 45 years of age who were familiar/experienced with clinically accepTable toothbrush and dentifrice usage and technique were considered for the study. Informed consent was obtained from the subjects after the rationale and purpose of the study were explained. Dentinal hypersensitivity was diagnosed based on the patient’s primary complaint and a comprehensive history about the patient’s perceived sensitivity to thermal stimuli [hot/cold], sweet/sour foods, beverages, and tooth brushing. Any possible causes of dental pain [caries/periodontal origin] were ruled out during clinical examination. The dentition considered for this investigation had no dental restorations; also, individuals with orthodontic appliances or bridgework that would be detrimental with the evaluation were excluded.

DH was assessed by air blast stimulation [a blast of air from a three-way syringe, connected via an air compressor at a pressure of 60 psi in room temperature]. The air jet was directed at the selected surface of the patient’s tooth for about 1 second from a distance of 1 cm from the selected Any uncomfortable sensation produced by the air blast stimulus was recorded This stimulus was accounted as a combination of thermal and evaporative stimuli (21). In each patient the examiner tooth that was most sensitive to the air blast stimulus was selected. Evaluation of DH was based on the patient’s subjective answer, using the visual analog scale [VAS] (22). Ordinal values from 0 to 10 on opposite ends of this scale represented “no pain/absence of pain” [value = 0] and “unbearable pain” [value =10]. The patients were requested to specify a value from 0 to 10 that best characterized their perceived level of pain.

To define the effectiveness DH therapy in this study, the following values were classified: Excellent [DH value of 0=no pain/ absence of pain]; Good [DH values 1-3=mild pain]; Unsatisfactory [DH values 4-6 = moderate pain]; Bad [DH values 7-9 =strong pain]; Unbearable [DH value 10=unbearable pain].

Generally, at every value>0 the pain was believed to be tolerable by the patients. A classification of “bad” was used when the final DH value was higher than the initial pain and the pain was not tolerated.

The participants were divided equally [n=15] into 5 groups: Group 1 treated with DL; Group 2 advised to use potassium nitrate toothpaste; Group 3 advised to use potassium nitrate mouthwash; Group 4 advised to use herbal toothpaste and Group 5 advised to use herbal mouthwash.

For the DL treatment in Group 1, the tooth was gently dried with a cotton roll before applying the laser. A Ga-Al-As 940 nm DL [Elaze 940, Biolase Inc., Irvine, CA, USA] was used as per the manufacturer’s instructions in a pulsed, defocused operation mode. Both the operator and the subjects used appropriate protective eye wear during the application. The power was set at 1.2- 1.5 W with the pulse duration of 0.20 seconds and pulse interval of 0.20 seconds. The ensure time per application was 15 seconds. Energy per application was 19 J.

The DL was applied perpendicular to the long axis of the tooth in a non-contact mode, point by point (23). Participants in the toothpaste groups were instructed to use the prescribed commercial brands of the respective toothpastes, and to brush twice daily for a period of 4 weeks. Participants in the mouthwash groups, were instructed to take 10 ml of the respective mouthwash, and rinse the mouth thoroughly for 30 seconds and expel, twice daily for 4 weeks respectively. Participants were told to restrict themselves to the prescribed products as the only treatment for their sensitive tooth [resorting to other products during the study period was not allowed]. Each group was recalled at weekly intervals for one consecutive weeks, and at the end of one month. Hypersensitivity scores were recorded before and after the therapy using VAS at all the follow up visits.

-Statistical analyses.

Sample size calculation was done to detect difference in reduction in VAS scores between the five groups at different time points using a two tailed significance level of 5% with a 90% power. The normality of the distributions was analysed using the Kolmogorov-Smirnov test. Comparisons among the groups and between the groups were tested. Kruskal-Wallis ANOVA was applied to compare VAS scores and change in the VAS scores at baseline, 1 week and 1 month among the five groups. Pair-wise comparisons of VAS scores between five groups was done by Mann-Whitney U- test. Wilcoxon matched pairs test by ranks was used for comparison of baseline, 1 week and 1month VAS scores in the five groups. The *p-value* was set at ˂0.05. The SPSS [IBM, Armonk, NY, USA] software was used for the analyses.

## Results

The data was expressed as Mean ± standard deviation [SD]. The normality of the distributions was analysed using the Kolmogorov-Smirnov test. Seventy-five participants [43 males and 32 females, age in years 34.22±5.86] were involved in this investigation. Comparisons among the groups and between the groups were tested. Comparison of the five groups with respect to VAS scores and change in the VAS scores at baseline, 1 week and 1 month was done by Kruskal Wallis ANOVA and changes from baseline to 1 week, from baseline to 1 month and from 1 week to 1 month was done by Wilcoxon matched pairs test (Table 1). Pair-wise comparisons between five groups [1, 2, 3, 4, 5] with respect to VAS scores was done by Mann-Whitney U test. Significant differences were observed at 1 week for groups 1 versus 4, and 2 versus 3,4 and 5. The pair-wise comparison was significant for groups 1 versus 3 and 4 (baseline to 1 month changes in VAS scores) and for groups 1 versus 2 and 2 versus 4 and 5(1 week to 1month changes in VAS scores).

**Table 1:**
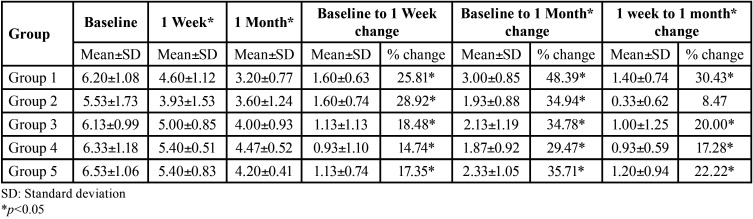
Comparison of five groups with respect to change in VAS scores at baseline, 1 week and 1 month and from baseline to 1 week, baseline to 1 month, 1 week to 1 month by Kruskal Wallis/Wilcoxon matched pairs test.

Statistical significant differences in the VAS scores were found in all the groups showing significant reduction of the VAS scores at 1week and 1month for all the therapeutics used when compared to baseline. Significant VAS reduction were seen at one week with the following observations: potassium nitrate tooth paste was better than laser treatment; laser treatment is better than herbal mouth wash and potassium nitrate mouthwash is better than potassium nitrate tooth paste and herbal tooth paste. Significant VAS reduction were seen at one month with the following observations: Laser is better than herbal mouth wash, potassium nitrate tooth paste and herbal tooth paste. Statistical significant difference in VAS score reduction was noticed for all the five groups when compared to baseline values compared with one month values, and when one week values were compared with one month values. Laser is better than herbal mouth wash and potassium nitrate tooth paste at one month compared with baseline. Laser is better than potassium nitrate mouth wash, potassium nitrate tooth paste is better than potassium nitrate mouth wash and herbal mouth wash is better than potassium nitrate mouth wash at one month when compared with one week. Laser, herbal mouth wash, potassium nitrate tooth paste and herbal tooth paste reduced DH at one week and one month compared with baseline and at one month when compared with one week. Potassium nitrate mouth wash reduced DH at one week and one month compared with baseline but was not significantly effective at one month compared with one week.

## Discussion

DH manifests as a pain response when dentin is exposed to thermal, chemical, and tactile stimuli. DH is a challenge for the clinician and a problem with many implications for the patient suffering from it (24). Home use products with active ingredients disrupting the hydrodynamic mechanism or blocking the neural transmission have been in use more often (25), and recently lasers have proven to be a proficient option for treating DH.

The current study compared commercially available, potassium nitrate and herbal toothpastes and mouthwashes, respectively, with DL Laser in the treatment of DH. DH has been shown to peak in ages 20-30 years and in mid-40s (6), hence an age range of 25-45 years was selected for the study. A parallel group design was chosen as recommended in the for the treatment of DH (26).

Numerous stimuli are thought to cause dentinal pain, but only some quantify as DH, thermal and air stimuli are suggested, but DH may still differ to various stimuli (27). Therefore, clinical studies should also evaluate changes in overall sensitivity to routine stimuli.

In this study the VAS was used to assess DH because of the ease by which it is understood by the patient and its sensitivity in discriminating amongst the effects of different treatment modalities, thus making it appropriate for evaluation (28).

Conventional DH treatment advocate the topical use of desensitizing agents, either professionally or at home such as dentinal tubule-occluding agents/sealants, protein precipitants and lately lasers are another in-office alternative for DH treatment, and has become a topic of research.

In our study, DL VAS showed significant reduction compared to other groups which is in accordance with the study conducted by Gerschman *et al*., (29), who observed that thermal and tactile sensitivity was reduced, respectively, in 67% and 65% of cases when the laser was applied to patients with hypersensitive teeth.

Potassium nitrate toothpaste showed better results than laser and other products with regard to VAS reduction at one week which can be explained by the report of Gomi *et al*., which verified that the efficacy of the treatment is directly proportional to an increase in the number of applications (30). In a six month follow up study, Aranha *et al*., compared a 660 nm DL to different desensitizing agents which concluded that the response of the laser was slower than the other agents initially (31), which is in agreement with our study.

The percentage change in VAS was better in DL when compared with other agents in the study at the end of one month. Also, our study indicates that potassium nitrate mouthwash reduced DH at one week and one month compared with baseline but was not significantly effective at one month compared with one week in comparison to potassium nitrate toothpaste and herbal products. The abrasive components of toothpaste can also bring about tubule occlusion. Since all the patients brushed with a fluoridated paste prior to rinsing with the allotted mouthwash, reduction in sensitivity due to brushing cannot be ruled out.

Despite these encouraging findings, it is interesting to note that a recent Cochrane database systemic review failed to find strong evidence supporting the efficacy of potassium nitrate formulations (11), and a latest literature review indicates lasers to be a promising, safe and beneficial mode of therapy for DH (32).

The herbal product employed in this investigation consisted of the following Cinnamomum zeylanicum, Syzygium aromaticum, Spinacia oleracea, Triphala, trikatu and Suryakshara [potassium nitrate]. The components responsible for reducing DH in the test group were Suryakshara and Spinacia oleracea. Each herbal formulation of mouth wash and paste consist of 30.0 mg Suryakshara [potassium nitrate] and 10.0 mg Spinacia oleracea per gram of dentifrice. It has been found that soluble oxalates and oxalic acid in phytocomplexes present in Spinacia oleracea form calcium oxalate crystals by reacting with dentinal calcium (20). Calcium oxalate crystals present in lyophilized phytocomplexes may penetrate inside dentinal tubules if their dimensions are less than 1-2 mm. The ratios of calcium and oxalate/calcium determine the effect of phytocomplexes on dentinal tubule occlusion. Low amounts of calcium and excess oxalate [oxalate/calcium ratio >1] induce binding of oxalate to calcium, producing calcium oxalate directly inside dentinal tubules. Oxalate crystals are small enough to penetrate the tubules and occlude tubular oriﬁces. A study has shown that treatment with oxalate-containing phytocomplexes induce microcrystal deposition on dentine and inside dentinal tubules and thus reduce the tubular diameters by forming crystals or crystal-like structures (14).

In the present study herbal mouth wash and tooth paste showed better change in VAS scores than the potassium nitrate mouth wash and paste when compared from baseline to one month. This can be explained by the fact that, as stated earlier, potassium nitrate lacks ability to occlude dentinal tubules, the Spinacia oleracea which is present in herbal mouth wash may have a possible mechanism of having a synergistic effect along with potassium nitrate in reducing DH by its dentinal tubule obliterating property. Herbal toothpaste like the one used in this study has been shown to be efficacious in the literature (33).

Although this short term study provided satisfactory results, the VAS scores post therapy could have been lower, especially with the DL group. However, the literature indicates that longer duration studies (34,35) have delivered such scores and one report (36) may not have reflected low VAS scores, but delivered clinically accepTable results.

## Conclusions

The results of this study indicates a role for DL as an in-office therapeutic procedure for DH. A combination of DL with home-use desensitizing agents may have a potential benefit in the treatment of DH.
